# The Role of Cross-Sectional Design and Orientation in Governing Energy Absorption of Additively Manufactured Polyamide 12 (PA12) Octet Lattices

**DOI:** 10.3390/polym17212817

**Published:** 2025-10-22

**Authors:** Muhammet Muaz Yalçın, Sedat İriç, Derya İriç, Mostafa S. A. Elsayed

**Affiliations:** 1 Mechanical Engineering, Sakarya University, 54050 Sakarya, Türkiye; siric@sakarya.edu.tr; 2 Sakarya University Technology Development Zones Management Inc. (Sakarya Teknokent), Sakarya University Esentepe Campus, 54050 Sakarya, Türkiye; 3 Arifiye Vocational School, Sakarya University of Applied Sciences, 54580 Arifiye, Türkiye; diric@subu.edu.tr; 4 Mechanical and Aerospace Engineering, Carleton University, Ottawa, ON K1S 5B6, Canada; mostafa.elsayed@carleton.ca

**Keywords:** multi jet fusion, lattice, PA12, cross-section effect, crashworthiness, strut orientation

## Abstract

This study investigates the influence of strut cross-sectional geometry and orientation on the crashworthiness of octet truss lattice structures produced via Multi Jet Fusion (MJF) using Polyamide 12 (PA12) material. All lattice configurations were designed and printed at a constant relative density of approximately 30%, ensuring equal mass and material usage across geometries. Quasi-static compression tests were conducted on lattices featuring circular, elliptical, rectangular, and square struts, with the latter two also evaluated at 0° and 90° orientations relative to the loading direction. Energy absorption (EA), specific energy absorption (SEA), crush force efficiency (CFE), and mean plateau stress metrics were employed to evaluate the structural energy absorption efficiency. The results highlight that strut geometry and orientation significantly alter mechanical behavior due to differences in moments of inertia. The circular strut lattice, used as the reference configuration, achieved an SEA of 0.79 J/g. Among the tested designs, the elliptical lattice exhibited the most pronounced variation: the non-rotated version showed the lowest SEA (0.63 J/g, ~20% lower than the reference), whereas the 90° rotated version yielded the highest SEA (0.92 J/g, ~16% higher). Rectangular struts displayed a similar trend, with rotated specimens outperforming their non-rotated counterparts. Square struts, however, showed negligible differences between orientations, as their rotational inertia remained constant. Overall, the findings demonstrate that optimizing strut cross-sections can enhance crashworthiness by improving energy dissipation and stabilizing deformation mechanisms under compressive loading. The rotated elliptical cross-section emerged as the most efficient configuration, offering superior SEA and crush stress efficiency. The findings highlight that cross-sectional design and orientation provide an effective mechanism for tuning mechanical performance in lightweight lattice materials without altering overall density or topology. These insights emphasize the potential of geometric tailoring in lattice design to meet safety and lightweight requirements in transportation, defense and biomedical applications.

## 1. Introduction

Lattice structures are increasingly significant in crashworthiness studies due to their unique mechanical properties and energy absorption characteristics. The interaction of these properties under different loading conditions is crucial in applications ranging from automotive to aerospace engineering. Various lattice configurations, including those optimized through topology and incorporating triply periodic minimal surfaces (TPMS), exhibit distinct energy absorption responses depending on factors such as strut design and material properties. The performance of lattice structures under axial loading has garnered specific interest [1,2,3,4,5,6,7,8,9,10]. Wang et al. investigated MTP-lattice structures, providing a comprehensive analysis of how strut patterns and geometric parameters influence crashworthiness indices, particularly energy absorption (EA) and specific energy absorption (SEA) [1]. Their findings indicate that the geometry of the lattice plays a pivotal role in maximizing energy dissipation during impact events. Similarly, Yao et al. [3] highlighted the stability of arch micro-strut lattice structures compared to straight configurations, noting the arch structure’s improved resistance to buckling under pressure, which enhances energy absorption performance. The literature suggests that structural defects and the inherent topology of lattice materials significantly affect energy absorption efficiency during extreme loading scenarios [4]. In terms of mechanical behavior, different lattice geometries respond variably to compressive forces, leading to distinct deformation mechanisms. Shi et al. [4] noted that a certain regime in lattice compression involves a linear elastic response followed by a plateau phase that characterizes energy storage before material densification occurs. This phase is critical for energy absorption performance, especially in crash scenarios. Notably, Zhao et al. [5] demonstrated that lattices based on TPMSs, specifically BCC structures, exhibit superior performance in SEA compared to traditional configurations, indicating a significant advantage in energy absorption characteristics. Moreover, the energy absorption capability is directly linked to the density and design of the cellular structure [6], with specific designs contributing to enhanced performance metrics.

The gradient of porosity within lattice structures can also lead to improved mechanical responses. Hybrid functionally graded structures have been shown to enhance energy absorption by aligning the design with anticipated stress distributions during impact [9]. Research indicates that such modifications can yield increases in energy absorption capacity by up to 110%, highlighting the impact of controlled accumulation of structural density on performance [10]. Furthermore, microengineering strategies, such as the introduction of surface defects and variations in strut thickness, have also been demonstrated to influence energy dissipation capabilities [11]. Additionally, the manufacturing process plays a crucial role in determining the performance characteristics of lattice structures [12,13,14,15,16,17,18]. Selective laser melting (SLM) techniques allow for precise control of lattice geometry, directly impacting mechanical attributes and energy absorption efficiency. Studies have shown that structures produced via SLM exhibit notable improvements in SEA, largely attributed to controlled porosity and enhanced material properties [12]. The integration of computer-aided design with advanced manufacturing techniques supports the optimization of lattice structures tailored to specific crashworthiness requirements. While a wide variety of lattice configurations—ranging from topology-optimized and TPMS-based structures to stochastic networks—have been investigated for their crashworthiness, the octet lattice has attracted particular attention due to its stretch-dominated load transfer mechanism and excellent strength-to-weight ratio. Building on these general insights into the mechanical behavior of cellular materials, the present study focuses on octet lattices to elucidate how strut cross-sectional design and orientation govern their axial energy absorption performance.

The axial compression behavior of octet lattice structures is critical for applications in lightweight design and crashworthiness. The octet lattice, characterized by its geometrical arrangement of elements, allows for efficient energy absorption and structural strength under compressive loads. A comprehensive understanding of the mechanical properties and failure mechanisms of these structures under axial compression has been established through various studies. One of the foundational works on the compressive behavior of octet lattices is provided by Li et al. [19], who detailed how the applied loads are primarily borne by the struts under tension and compression, while bending effects are secondary. This understanding is pivotal as it indicates that optimizing strut orientation could enhance the overall load-bearing capacity of the lattice. In experiments by Surjadi et al. [20], it was demonstrated that octet lattices exhibit stretching-dominated behavior during compression, which aligns with findings that suggest better performance in directionally loaded scenarios. Specifically, the study highlights the influence of design features in tailoring the response of octet lattices to enhance mechanical performance. The failure mechanisms of octet lattices under axial loading have been further examined. Després et al. [21] noted that lattice structures manufactured through SLM showed different failure characteristics when loaded axially compared to off-axis, confirming that strut alignment is crucial for maintaining structural integrity under load. Furthermore, studies have indicated the formation of diagonal shear bands during compression, leading to reduced load-bearing capacity over time [22]. This observation emphasizes the importance of understanding deformation patterns to mitigate premature failure. Wang et al. [1] contributed insights into how energy absorption capabilities are critical for the crashworthiness of these lattices, evaluating various metrics to determine optimal designs for specific applications. Their findings indicate that energy absorption correlates with both material properties and lattice geometry, signifying the necessity for a holistic approach in lattice design. Similarly, Adel’s [23] work highlights that the critical failure point typically occurs near nodes due to the concentrated stresses at these junctions, reaffirming the need to reinforce these areas to enhance load resistance.

Investigations into the SEA characteristics of octet lattices have been further advanced by Balan and Raj [10], who found that the absorptive capacity can increase significantly with optimized design parameters, achieving drastic enhancements in energy performance under compressive load. This aligns with earlier work by Zhao et al. [5] that identified the mechanical disadvantages of varying truss configurations and their energy efficiency under different loading conditions. Additionally, morphological considerations play a significant role in the mechanical behavior of octet lattices. Research by Zhao et al. [24] indicated that variations in lattice design lead to changes in mechanical properties under compression, stressing the importance of meticulous architectural choices in optimizing performance. Following these insights, the modeling of compressive behaviors continues to evolve, with recent studies like that of Kalia et al. [25] demonstrating that octet meta-structures can achieve high yield strengths, thus further widening their applications in structural engineering and beyond. As more advanced manufacturing techniques become available, such as additive manufacturing, the potential for tailoring octet lattice structures with intricate designs further augments their utility in various applications, from aerospace components to biomedical devices. The ongoing research into the mechanical properties and behaviors under axial compression lays a robust foundation for advancements in materials science and engineering. The crashworthiness of octet lattice structures is significantly influenced by the geometrical cross-section of the structural elements (struts) that comprise the lattice. Variations in cross-sectional shape and size directly alter the mechanical behavior of these lattices under dynamic loading conditions, influencing energy absorption and deformation characteristics during crash events. Research has shown that octet lattices with different cross-sectional geometries can exhibit varied mechanical properties. For example, Khan et al. [22] demonstrated that lattices with optimized cross-sectional designs can prevent shear band formation and maintain structural integrity during compressive loading, thereby enhancing crashworthiness. This aligns with the principle that the geometry of struts plays a crucial role in load distribution and energy absorption capacity. The authors posited that an ideal cross-section helps minimize stress concentrations, thereby enhancing structural performance during impact.

The crashworthiness of octet lattice structures is strongly governed by the geometrical design of the struts forming the lattice framework, where variations in cross-sectional shape and orientation critically affect load transfer mechanisms, deformation behavior, and energy absorption capacity during axial compression. Previous studies have shown that optimizing strut geometry can mitigate shear-band formation and improve structural integrity under compressive loading [22], while adjusting the strut aspect ratio and shape enhances specific energy absorption (SEA) and promotes more uniform stress distribution [1]. Similarly, Vangelatos et al. [26] demonstrated that modifying strut connectivity and introducing vacancies influence the torsional and axial stability of octet lattices, and Adel et al. [23] confirmed that failures often initiate at nodes due to stress concentration, which can be reduced by cross-sectional tailoring. Advances in additive manufacturing have further enabled precise manipulation of strut geometry for lightweight yet strong designs [5]. Despite these insights, prior works have focused mainly on topology-centric comparisons (e.g., BCC/FCC/OT lattices) or on global geometric parameters such as strut angle, thickness, or graded density, without isolating the influence of local cross-sectional shape and its orientation within a fixed octet topology [27,28]. In contrast, the present study experimentally isolates these effects by keeping topology and relative density constant while varying the cross-section (circle, ellipse, rectangle, square) and rotation (0°/90°) in MJF-printed PA12 octet lattices. This approach provides the first systematic experimental evidence of how local cross-sectional anisotropy and its rotation—at constant mass—govern the energy absorption (EA), specific energy absorption (SEA), and crush-stress efficiency (CSE) of PA12 octet structures, thereby filling a key gap in the literature [29,30].

While numerous studies have explored the influence of topology, density gradient, and material system on the mechanical response of lattice structures, limited attention has been directed toward the role of strut cross-sectional geometry and its orientation, particularly for polymer lattices produced via Multi Jet Fusion (MJF). Existing research on PA12 and octet lattices has primarily concentrated on global architecture and material behavior, leaving the effects of local strut morphology largely unexplored. In contrast, this study experimentally isolates and quantifies how variations in cross-sectional design and rotation govern the specific energy absorption (SEA) and crush-stress efficiency of PA12 octet lattices. By holding topology and relative density constant, it is demonstrated that tuning the local moment of inertia through cross-section shape and 0°/90° orientation substantially alters the energy absorption performance, thereby establishing a practical, material-agnostic pathway for enhancing crashworthiness.

This study uniquely explores how fundamental cross-sectional geometries—circular, elliptical, rectangular, and square—and their rotational orientations influence the energy absorption behavior of additively manufactured octet lattices. The rationale behind selecting these configurations lies in their distinct moments of inertia and symmetry properties, which enable systematic evaluation of geometry-induced anisotropy in load transfer and deformation. The findings offer new design insights into the geometric optimization of polymeric lattices for lightweight and crashworthy applications.

## 2. Materials and Methods

### 2.1. Lattice Parent Material

The lattice structures were fabricated from HP 3D High Reusability Polyamide 12 (PA12) (HP, Istanbul, Türkiye), a thermoplastic powder used to produce high-density parts with balanced mechanical properties. HP 3D High Reusability PA 12 provides good chemical resistance to oils, greases, aliphatic hydrocarbons, and alkalies, and is the optimal material for complex assemblies, housings, enclosures, and watertight applications. Due to the surplus powder reusability, the waste can be minimized, thus achieving a low cost per part. The general mechanical properties of the HP 3D High Reusability PA12 powder material are summarized in Table 1.

### 2.2. Design and Geometry of Lattice Structures

Each specimen consisted of 3 × 3 × 1 octet unit cells (10 mm per side), resulting in an overall sample size of 30 × 30 × 10 mm^3^. This scale was selected to minimize size effects while maintaining experimental practicality. All configurations shared identical external dimensions and contact boundaries, ensuring consistent boundary conditions across tests. The reported mechanical responses, therefore, represent the meso-scale behavior of the lattice and are not intended for direct upscaling without further analysis.

**Figure 1:**
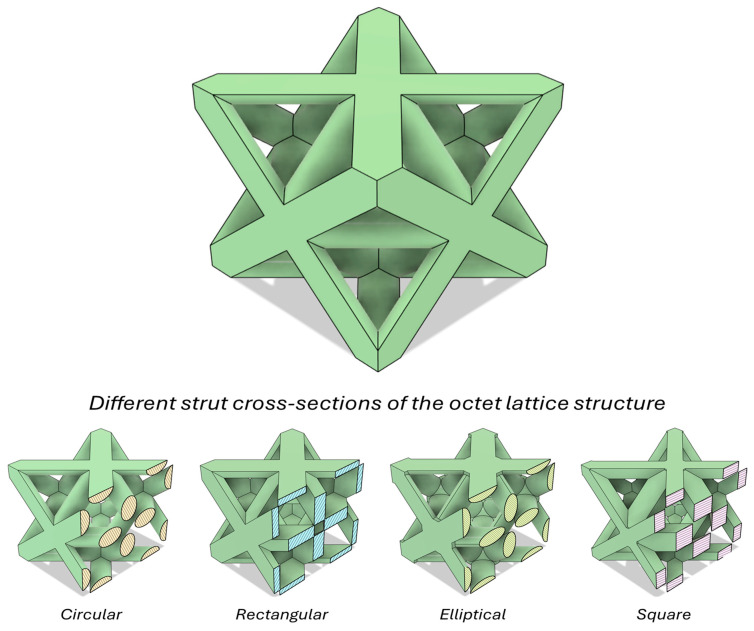
Schematic view of an octet unit cell lattice: different strut cross-section geometries and their clockwise rotated configurations.

The octet lattice structures were produced with different cross-sections, such as square, rectangular, and elliptical cross-sections, to determine the cross-section effect on the axial compressive behavior of lattice structures. Additionally, these different cross-sections were rotated 90° clockwise to observe the effect of the strut orientation as well. Circular strut cross-section was used as the reference strut geometry to compare the other cross-sections. In naming the samples, the rotation orientation of the strut is indicated by using 0° and 90° extensions after the strut cross-section geometry. If no rotation was applied to the strut, 0° is used; if rotation was applied, the 90° extension is added. Only the rotated version of the square sample is named diamond, without using any angle extension. All specimens were fabricated at 30% relative density, taking into account the production capability of the printer and the authors’ previous studies [2,18], resulting in nearly identical masses (minor deviations only). Three samples were printed for each different cross-sectioned lattice structures. The octet lattices with circular cross-section had a strut diameter of 0.87 mm [7,8,18]. A sample view of octet topology and different cross-section views were demonstrated in Figure 1, which is given below.

### 2.3. Fabrication of Specimens

The specimens were produced using an HP Jet Fusion 4000 (HP, Istanbul, Türkiye) 3D printer employing the Multi Jet Fusion (MJF) process developed by Hewlett-Packard. In this technique, layers of PA12 powder are selectively fused by applying fusing and detailing agents, followed by infrared energy exposure, enabling accurate reproduction of fine strut geometries.

All specimens were fabricated in a single, consistent build orientation (layers parallel to the build plate) to eliminate orientation as a confounding variable; assessing orientation-dependent anisotropy of MJF-PA12 is beyond the scope of this work and is reserved for future studies.

The MJF process provides high geometric fidelity and uniform density, making it suitable for small-scale production and customized lattice designs. The printing orientation was chosen to minimize residual stresses and ensure dimensional consistency across all samples. After printing, the specimens were air-cooled, depowdered, and cleaned before mechanical testing.

### 2.4. Experimental Testing Set-Up

Quasi-static compression tests were performed using an MTS universal testing machine (MTS, Istanbul, Türkiye) equipped with a 25 kN-load cell, which provided sufficient accuracy and resolution for the measured force range. The tests were conducted under displacement-controlled loading, with the crosshead velocity set to 0.01 mm/s, corresponding to an average engineering strain rate of 0.001 s^−1^ based on the initial specimen height (10 mm). The experimental configuration and measurement protocol followed the same procedure as described in our earlier work [7,8,18], adapted here for PA12 octet lattices.

**Figure 2:**
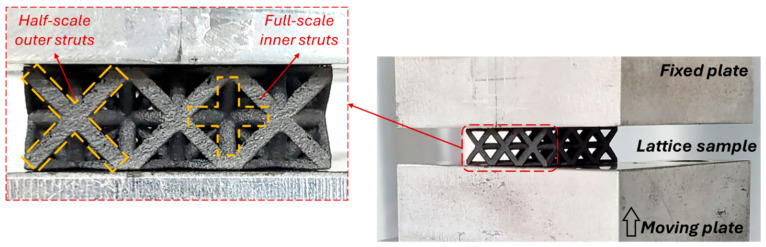
The overall view of the axial compression test setup and a detailed front view of the octet lattice with circular cross-sectioned strut.

Owing to the small size and open-cell nature of the lattices, strain could not be directly measured using an extensometer. Instead, the crosshead displacement recorded by the MTS controller was used to determine the global strain, a standard practice for quasi-static testing of cellular or lattice materials where local strain measurement is impractical. The MTS data-acquisition system continuously recorded load–displacement data, which was subsequently converted to stress–strain curves. Figure 2 shows the compression test setup and the detailed front view of the octet lattice.

Each configuration was tested in triplicate to ensure repeatability. The mean response of the three tests was used for subsequent stress–strain and energy-efficiency analyses.

Each compression test was continued until the specimen reached complete densification, identified by the onset of a steep stress rise following the plateau stage in the stress–strain curve. Testing was automatically stopped when either the densification limit was reached or the crosshead displacement exceeded 8 mm to prevent over-travel of the actuator.

### 2.5. Assessment of Crashworthiness Criteria

Determining appropriate crashworthiness indicators is essential for evaluating a structure’s impact performance. Data collected from the experimental analyses included the following crashworthiness parameters, the calculation of which allows for quantifying the performance of lattice materials, in addition to stress–strain and efficiency–strain data throughout the compression event. Energy Absorption Efficiency, also “efficiency” or η, is calculated from the stress–strain (σ-ε) curve as follows:

(1) $$\eta \left(\varepsilon \right)=\frac{1}{\sigma (\varepsilon )}\int_{0}^{\varepsilon }\sigma \left(\varepsilon \right)d\varepsilon$$

where the stress is obtained from the overall compressive force divided by the surface area of the upper surface of the lattice unit cell envelope (not the surface area of the lattice structures that the upper plate touches), and the strain is obtained from the displacement of the uppermost surface of the lattice divided by the original lattice unit cell height, a method utilized in [39,40].

Densification Strain: Per the energy absorption efficiency method, the densification strain, ε_D_, is as follows:

(2) $${\frac{d\eta (\varepsilon )\;\;\;\;}{\;\;\;\;\;\;d\varepsilon \;\;\;\;\;}|}_{\varepsilon =\varepsilon_{D}}=0$$

which is the strain at the maximum efficiency point [41,42].

Two parameters—energy absorption (EA) and specific energy absorption (SEA)—were proposed to qualitatively examine the crashworthiness of the lattice structures under axial loading performance, as the literature [43,44,45,46] indicates. Two indications were employed in this study to evaluate auxetic beam crashworthiness. The *EA* indicates the energy absorbed by the lattice beam structure for a specific displacement value, considering the force–displacement curve. Hence, the *EA* can be explained as follows:

(3) $$\mathrm{EA}=\int_{0}^{d}F\left(y\right)\mathrm{dy}$$

where *F*(*y*) is the instantaneous load carried by the beam structure and *d* is the compression displacement. The SEA, described as the energy absorbed per unit mass, has been broadly used as follows:

(4) $$\mathrm{SEA}=\frac{\mathrm{EA}}{m}$$

where *m* is the mass of the lattice beam structure and is calculated for the length between the fixed supports.

In addition to energy absorption (EA) and specific energy absorption (SEA), the Crush Stress Efficiency (CSE) was calculated to assess the uniformity of load-bearing behavior during compression. The CSE is defined as the ratio of the mean compressive stress (σ_mean_) to the maximum stress (σ_max_):

(5) $$\mathrm{CSE}=\frac{\sigma_{mean}}{\sigma_{max}}\;$$

A higher CSE value indicates a more stable energy absorption response and reduced stress fluctuations, signifying a more efficient crashworthy structure.

### 2.6. Second Moment of Area and Member Stability

To rationalize the effect of strut cross-section shape and orientation on the compressive response of the octet lattice, the relationship between the second moment of area and the critical buckling load is examined. For a slender strut of length L, elastic modulus E, and pinned–pinned boundary conditions, the Euler buckling load is:

(6) $$P_{cr}=\frac{\pi^{2}\;E\;I_{min}}{L^{2}}$$

where I_min_ is the minimum principal second moment of area about the centroidal axis.

The expressions for I of various strut cross-sections are:

Circular cross-section (diameter d):

(7a) $$I=\frac{\pi \;d^{4}}{64}$$

Elliptical cross-section (major axis a, minor axis b):

(7b) $$I_{x}=\frac{\pi \;a\;b^{3}}{4}$$

(7c) $$I_{y}=\frac{\pi \;a^{3}\;b}{4}$$

Rectangular cross-section (width b, height h):

(7d) $$I_{x}=\frac{b\;h^{3}}{12}$$

(7e) $$I_{y}=\frac{h\;b^{3}}{12}$$

Square cross-section (side s):

(7f) $$I=\frac{s^{4}}{12}$$

- Orientation Effect

For elliptical or rectangular members, rotation by 90° interchanges I_x_ and I_y_. Defining the moment of inertia ratio as:

(8) $$R_{I}=\frac{I_{max}}{I_{min}}$$

The ratio of the critical buckling loads for the two orientations becomes:

(9) $$\frac{P_{cr,90}}{P_{cr,0}}=R_{I}$$

For a representative aspect ratio (a/b) = 2:

(10) $$R_{I}={\frac{a}{b}}^{2}=4$$

Hence, the rotated configuration (major axis aligned with the loading direction) theoretically supports up to four times higher Euler buckling load, consistent with the experimentally observed 15–20% increase in plateau stress and specific energy absorption (SEA).

- Implications for Lattice Performance

Since the octet lattice stiffness scales with the axial stiffness of its struts and collapse initiates via member buckling, the specific energy absorption can be approximately correlated with the minimum moment of inertia as:

(11) $$SEA\propto {I_{min}}^{\frac{1}{2}}$$

Therefore, modest geometric changes in cross-sectional aspect ratio—while keeping relative density constant—can yield measurable improvements in both plateau stress and energy absorption efficiency.

## 3. Results and Discussion

In the graphs, only the data representing the average value of the test results for the sample were used. The main reason for this is that two different curves, stress and efficiency, are used for each sample in the graphs, and there is concern that visual confusion may arise if repeated test data is used. The force–displacement data obtained from the tests were converted into stress–strain curves. Efficiency curves were also obtained accordingly.

### 3.1. Circular Cross-Section

**Figure 3:**
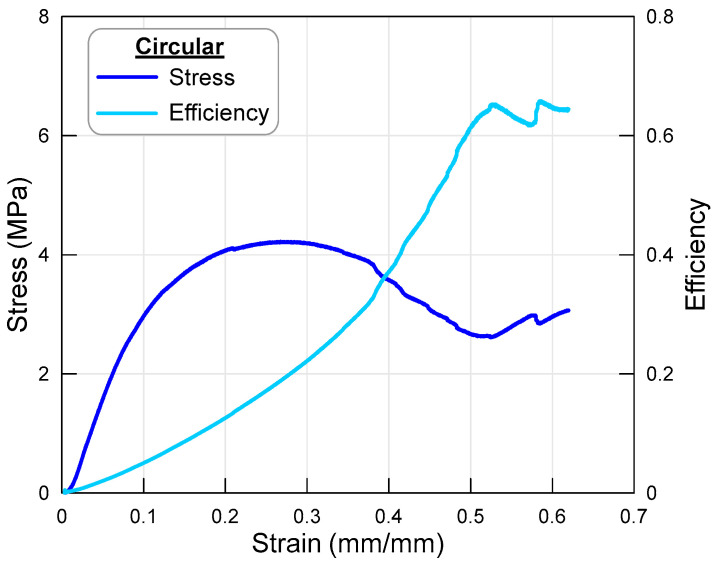
Stress–strain and efficiency–strain curves of the octet lattice structure with circular cross-sectioned strut.

The following graph shows the stress–strain curves and efficiency curves of an octet lattice structure with circular struts under axial load. In Figure 3 below, the dark blue color represents the stress curve, while the light blue color represents the efficiency curve. Stress data was obtained by dividing the force data by the surface area of the sample, while strain data was obtained by dividing the compression displacement values by the initial height of the sample.

**Figure 4:**
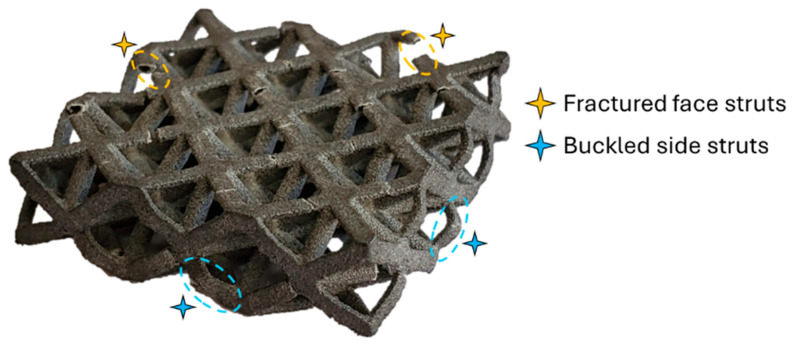
Axially crushed octet lattice with circular cross-section.

As shown in Figure 3, the stress value increased rapidly at the beginning of the test, reaching approximately 3 MPa, after which the rate of increase slowed down, and the curve flattened out at around 4 MPa (the highest value on the stress curve). It is also possible to mention that the elastic region continues to approximately 0.04 strain value. The highest stress value of 4 MPa is also important as it indicates the load-bearing capacity of the lattice structure. Subsequently, the curve enters a downward trend due to the decrease in the load-bearing capacity of the lattice structure. Here, it can be said that the struts lose their load-bearing capacity due to deformation at the struts of the lattice structure. It can be stated that the deformation dominating the decrease in load-bearing capacity is particularly buckling at the struts connecting the upper and lower surfaces (the sections marked with blue stars in Figure 4). As can be seen from the post-test sample image given in Figure 3, damage in the form of fractures has also occurred at the surface struts. When examining the efficiency curve of the specimen, it can be seen that the curve continuously increases until it reaches its approximate maximum value. Since the maximum efficiency value also indicates the load-bearing capacity limit of the lattice specimen (beginning of the densification region), the energy value that the specimen can absorb was calculated accordingly.

### 3.2. Elliptical Cross-Section

**Figure 5:**
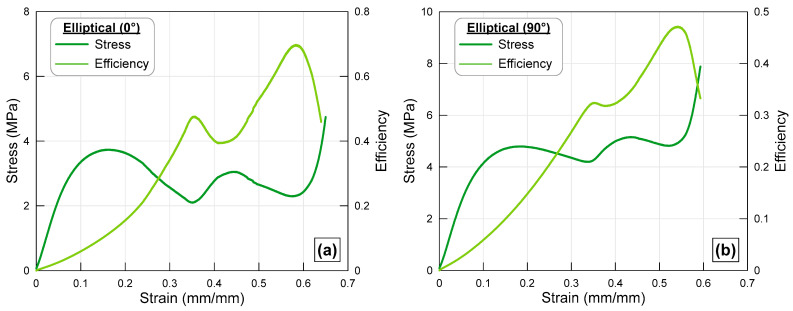
Stress–strain and efficiency–strain curves of the octet lattice structure with (**a**) elliptical cross-sectioned strut and (**b**) 90° rotated elliptical cross-sectioned strut.

Figure 5 shows the stress–strain and efficiency–strain curves for an octet structure with elliptical cross-section struts under axial compression. The struts in Figure 5b belong to the specimen rotated 90° clockwise relative to the struts given in Figure 5a. When comparing the stress–strain curves of the specimens, the general characteristics of the curves are quite different at first observation. Indeed, while the maximum stress value reached by the normal elliptical strut is around 4 MPa, the stress value in the 90° rotated elliptical struts has increased by approximately 25% to reach the 5 MPa level. It is also noteworthy that the stress curve of the lattice structure obtained by rotating the struts oscillates within a lower range compared to the original strut specimen. This difference in the stress curve directly affects the efficiency curves of the specimens. Due to this situation, the value at which the efficiency curve reaches its maximum value has decreased from approximately 0.58 in the normal elliptical strut lattice structure to 0.54. This situation is a notable change in that it causes a slight decrease in the energy absorbed by the lattice with rotated elliptical struts. The higher stress values in lattices with rotated struts can be explained by the change in the moment of inertia of the struts, even though they have the same cross-sectional area. Indeed, the moment of inertia values of normal elliptical struts are much lower than those of 90° rotated struts. It can be said that this change in the moment of inertia of the struts has directly affected the load-bearing capacity of the lattice structures. The sharp stress increase observed at the final stage of compression corresponds to the densification of the lattice, where the collapse of pores and inter-strut voids causes a rapid rise in load-bearing capacity due to the transition from cellular to solid-like deformation.

### 3.3. Rectangular Cross-Section

**Figure 6:**
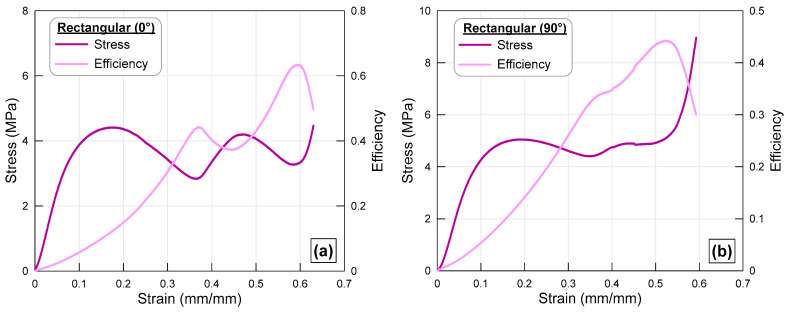
Stress–strain and efficiency–strain curves of the octet lattice structure with (**a**) rectangular cross-sectioned strut and (**b**) 90° turned rectangular cross-sectioned strut.

The curves showing the change in stress and efficiency values of the octet structure with rectangular cross-section struts under axial compression as a function of strain are shown together in Figure 6. Since all lattices have the same relative density (30%), their total masses and strut cross-sectional areas are nearly identical, with minor variations (<2%) arising from the modeling and printing resolution of the MJF process. Therefore, despite having equal cross-sectional areas, the fact that the struts have different geometries has a very noticeable effect on the load-bearing capacity of the lattice structures. A similar stress difference to that seen in elliptical cross-section specimens also appeared in the curves of trusses with rectangular cross-section struts. However, the stress values in these specimens are higher than those with elliptical cross-sections in both cases (rotated and normal struts). Another noteworthy point is that the lattice with 90° rotated rectangular cross-section struts has a more stable stress curve compared to its elliptical cross-section counterparts. Additionally, the difference in strain values at which the efficiency curves reach their maximum is greater than that of the elliptical cross-section counterparts. As seen in Figure 6b, the lattice specimen with 90° rotated struts reached its load-carrying capacity limit earlier than the other lattice specimen. Although this indicates that the useful load carried by the specimen in the axial crushing condition will end at lower displacements, the increase in stress values of the specimen is at a level that tolerates the resulting loss.

### 3.4. Square Cross-Section

**Figure 7:**
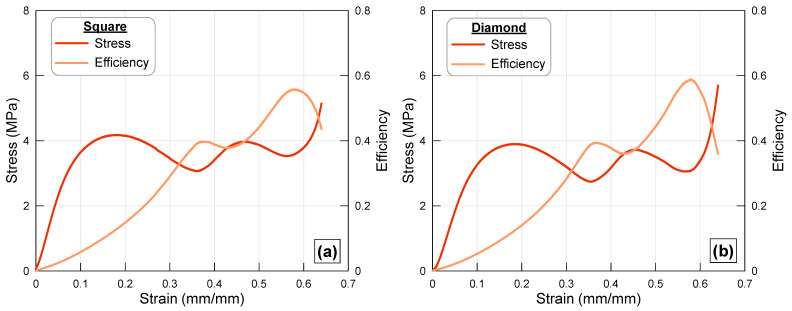
Stress–strain and efficiency–strain curves of the octet lattice structure with (**a**) square cross-sectioned strut and (**b**) diamond elliptical cross-sectioned strut.

The stress–strain and efficiency–strain curves for octet lattice structures with square cross-sections and square cross-sections rotated clockwise are shown together in Figure 7. The first notable feature is that the force curves in these specimens are quite close to each other, unlike those in other specimens (rectangular and elliptical cross-sections). It was mentioned above that the difference in stress values is directly related to the cross-sectional moments of inertia of the struts. It is a fact that the moment of inertia of a square cross-section member does not change with rotation because its center of mass and axes remain the same, and thus the Mohr circle is at a single point. Therefore, the fact that stress remains at similar values for both types of truss (Figure 7) can be attributed to this. Indeed, the differences in sectional moments of inertia resulting from the rotation of rectangular and elliptical struts are approximately 4-fold. As a natural consequence, the load-bearing capacities of the frames change significantly (Figure 5 and Figure 6). The fact that the stress values are both approximately the same and have similar characteristics has led to the strain value at which the efficiency curve reaches its maximum value also being similar.

### 3.5. Discussion of Comparative Results

Table 2 below contains the results of the crushing energy efficiency parameters for all samples used in the experimental study. Absorbed energy, specific energy absorption, and crushing stress efficiency parameters were used to compare the energy efficiency of lattice structures. Indeed, these parameters are frequently used in similar studies in the literature [39,47,48,49]. The SEA value of the circular lattice, used as the reference geometry, was 0.79 J/g. Depending on the cross-sectional configuration, some lattices exhibited lower SEA values—indicating reduced energy absorption efficiency—while others exceeded this reference, confirming that the crashworthiness of octet lattices is highly dependent on strut geometry and orientation. The lowest SEA value of 0.63 J/g was obtained in the elliptical cross-section lattice structure. This value is approximately 20% lower than the reference value. The highest SEA value of 0.92 J/g, approximately 16% higher than the reference value, was obtained in the sample with rotated elliptical struts. In the rectangular cross-section sample group, higher SEA values than the reference value were obtained for both strut types (normal and clockwise-rotated struts). It is noteworthy that the lattice with struts in their normal (0° non-rotated) orientation absorbs less energy than the lattice with struts rotated by 90°, owing to the corresponding change in the struts’ moment of inertia. As explained in the previous section, this can be explained directly by the struts’ moment of inertia. Indeed, considering that the clockwise rotated state of the struts has a moment of inertia four times higher than the normal state, the difference in energy values can be more easily understood. The crush stress efficiency parameter, frequently used in energy-absorbing structures, is obtained as the ratio of the average stress value of the sample to the maximum stress value. It can be said that the closer this parameter is to 1, the closer it is to an ideal energy-absorbing structure. When the samples are examined in terms of this parameter, it can be seen that the highest value is obtained in octet lattices with rotated rectangular and elliptical struts. When all parameters are examined in terms of energy efficiency, it can be said that the most efficient sample is the octet lattice with clockwise-rotated elliptical struts.

While a comprehensive analysis of the deformation behavior of the specimens is not feasible due to the purely experimental design of this study, meaningful insights can be derived regarding the deformation characteristics associated with different strut cross-sections. This can be achieved by carefully examining the stress–strain curves presented above, in conjunction with the data detailed in Table 2. In the circular and square cross-sectioned strut lattices, deformation was governed primarily by progressive strut buckling at the mid-span regions, with limited fracture observed near the nodes. The geometric symmetry of these sections promoted uniform load paths and resulted in smooth, stable stress–strain responses. In contrast, the elliptical and rectangular lattices—particularly in their non-rotated (0°) configurations—exhibited early localized buckling driven by anisotropic bending stiffness, producing oscillatory stress–strain curves. The higher curvature differences between their major and minor axes amplified instability when the weaker axis aligned with the compressive direction. Conversely, the rotated (90°) variants displayed markedly more stable mechanical behavior, as reorientation of the principal inertia axis redistributed compressive loads and delayed local buckling. These rotated elliptical and rectangular struts developed smeared deformation zones instead of discrete hinge points, enabling more uniform strain distribution and gradual energy dissipation throughout the structure.

The observed performance trends were consistent across all three replicates, with small standard deviations demonstrating high repeatability. The superior specific energy absorption (SEA) of the rotated elliptical and rectangular lattices was reproducibly observed in every test.

Previous research on PA12-based lattice structures provides valuable context for interpreting the SEA values obtained in this study. Khiavi and Sadeghi [27] systematically investigated the effect of topology on the strength and energy absorption of PA12 non-auxetic strut-based lattice structures and reported that the FBCC_z_ topology achieved an energy absorption of approximately 0.281 J/g, underscoring how geometric configuration significantly affects crashworthiness performance. In comparison, the SEA values measured here for octet lattices with different cross-sectional geometries (ranging from 0.63 to 0.92 J/g) are of a similar magnitude, reinforcing the strong dependence of energy dissipation on geometric tailoring. Likewise, Cronau et al. [50], in 2025, examined the energy absorption of 3D-printed stochastic lattice structures made from PA12 under dynamic loading conditions. Their tests yielded SEA values between 0.35 and 0.40 J/g for relative densities around 25%, demonstrating that strain-rate effects can further enhance the energy absorption capacity of PA12 lattices. These findings complement the present results and suggest that including dynamic or high-strain-rate experiments in future work would extend the applicability of the conclusions to real crash scenarios.

Additional comparative insights can be drawn from recent polymer lattice studies. Schneider et al. [51] conducted a comparative performance evaluation of microarchitected polymer lattices and reported that PA12 gyroid lattices with around 23% relative density achieved an energy absorption efficiency of about 68%, highlighting the crucial role of topology in optimizing crashworthiness. Similarly, in their study Nasim and Galvanetto [52], mechanical characterization of additively manufactured PA12 lattice structures under quasi-static compression presented baseline SEA values under quasi-static loading for various PA12 geometries, indicating that geometric imperfections and process-induced anisotropy can limit achievable SEA in practice.

Taken together, these studies help to position the current results within the broader PA12 lattice literature. They confirm that the enhanced SEA and crush-stress efficiency observed here—especially for the rotated elliptical and rectangular struts—are consistent with general trends reported for PA12 lattices, while simultaneously emphasizing the unique contribution of cross-sectional design and strut orientation optimization to crashworthy lightweight structures.

While the present study does not include a systematic investigation of build-orientation effects, it was intentionally designed to isolate the influence of strut cross-section geometry and its rotation under otherwise identical manufacturing conditions. To ensure that the observed differences in energy absorption and deformation behavior were purely geometry-driven, all specimens were fabricated with the same build orientation—layers parallel to the build plate—thereby avoiding confounding effects from process-induced anisotropy. Nonetheless, prior studies have demonstrated that build orientation can influence the mechanical response of MJF-printed polymers to varying degrees. For instance, Osswald et al. [53] introduced anisotropy-aware failure criteria for MJF-PA12 components, and Calignano et al. [54] reported measurable yet relatively moderate orientation-dependent variations in stiffness and fracture behavior when comparing X/Y/Z orientations in MJF and SLS. Similarly, it was observed that tensile strength sensitivity to orientation in MJF-PA12 can be limited and dataset-dependent [55], while Khorasani et al. [56] summarized that MJF generally produces lower porosity and reduced anisotropy than SLS but still warrants dedicated experimental evaluation.

## 4. Conclusions

This study experimentally demonstrates that the cross-sectional geometry and orientation of struts play a decisive role in determining the crashworthiness of PA12 octet lattices fabricated via Multi Jet Fusion. By isolating geometric influences from material and density effects, the work introduces a novel, geometry-based approach to optimize energy absorption. Variations in the strut’s moment of inertia—arising from differences in cross-sectional shape and rotation—produced marked changes in load-bearing capacity, deformation stability, and specific energy absorption (SEA). In particular, rotated elliptical and rectangular struts exhibited improved resistance to buckling and smoother, more stable stress–strain responses, achieving SEA values up to 0.92 J/g. These results establish a practical framework for designing next-generation lightweight lattice cores with superior crash performance and manufacturability.

Furthermore, the findings provide clear evidence that cross-sectional anisotropy and its rotation—at constant mass—can lead to meaningful improvements in both SEA and crush-stress efficiency (CSE). Unlike topology alterations or material upgrades, this represents a simple, low-cost geometric lever that can be readily implemented in lightweight, crashworthy structures. Even within a single material system such as PA12, such geometric tailoring yields performance gains comparable to those achieved through material enhancement, underscoring its potential for use in impact absorbers, structural cores, and biomedical scaffold applications where both efficiency and manufacturability are critical.

Future research should expand on these findings by (i) incorporating dynamic and high strain-rate loading to replicate real crash conditions; (ii) developing validated finite element models to predict deformation mechanisms; (iii) exploring hybrid or graded lattice architectures that combine multiple cross-section types or materials. Integrating these approaches will enable the design of next-generation lattice systems that balance low weight, high energy dissipation, and structural reliability.

## Figures and Tables

**Table 1:** Mechanical and physical properties of HP 3D High Reusability PA 12 powder [31].

Category	Measurement	Value	Method
**General properties**	Powder melting point (DSC)	187 °C	ASTM D3418 [32]
	Particle size	60 µm	ASTM D3451 [33]
	Bulk density of powder	0.425 g/cm^3^	ASTM D1895 [34]
	Density of parts	1.01 g/cm^3^	ASTM D792 [35]
**Mechanical properties**	Tensile strength, max load, XY	48 MPa	ASTM D638 [36]
	Tensile strength, max load, Z	48 MPa	ASTM D638 [36]
	Tensile modulus, XY	1700 MPa	ASTM D638 [36]
	Tensile modulus, Z	1800 MPa	ASTM D638 [36]
	Elongation at break, XY	20%	ASTM D638 [36]
	Elongation at break, Z	15%	ASTM D638 [36]
	Flexural strength (@ 5%), XY	65 MPa	ASTM D790 [37]
	Flexural strength (@ 5%), Z	70 MPa	ASTM D790 [37]
	Flexural modulus, XY	1730 MPa	ASTM D790 [37]
	Flexural modulus, Z	1730 MPa	ASTM D790 [37]
	Izod impact notched (@ 3.2 mm, 23 °C), XYZ	3.5 kJ/m^2^	ASTM D256 [38]

**Table 2:** Crashworthiness parameters of octet lattice structures with different strut cross-section geometries.

Strut Geometry	Densification Strain (mm/mm)	Mass (g)	σ_mean_	σ_max_	Energy Absorbed	CSE	SEA
			(MPa)	(MPa)	(J)	(MPa/MPa)	(J/g)
**Circular**	0.62	2.5	3.24	4.22	2.01	0.77	0.79
***S.D.***	*±0.01*	*±0.01*	*±0.00*	*±0.02*	*±0.03*	*±0.01*	*±0.03*
**Elliptical_0°**	0.59	2.5	2.74	3.74	1.62	0.74	0.63
***S.D.***	*±0.01*	*±0.01*	*±0.00*	*±0.03*	*±0.03*	*±0.01*	*±0.03*
**Elliptical_90°**	0.55	2.5	4.29	5.16	2.36	0.84	0.92
***S.D.***	*±0.01*	*±0.01*	*±0.01*	*±0.02*	*±0.04*	*±0.01*	*±0.04*
**Rectangular_0°**	0.6	2.5	3.52	4.41	2.11	0.8	0.82
***S.D.***	*±0.01*	*±0.00*	*±0.01*	*±0.02*	*±0.03*	*±0.01*	*±0.03*
**Rectangular_90°**	0.53	2.5	4.36	5.19	2.31	0.84	0.9
***S.D.***	*±0.01*	*±0.00*	*±0.01*	*±0.02*	*±0.04*	*±0.01*	*±0.04*
**Square**	0.58	2.5	3.48	4.18	2.02	0.83	0.79
***S.D.***	*±0.01*	*±0.00*	*±0.01*	*±0.03*	*±0.03*	*±0.01*	*±0.03*
**Diamond**	0.59	2.5	3.1	3.9	1.83	0.8	0.72
***S.D.***	*±0.01*	*±0.00*	*±0.01*	*±0.03*	*±0.03*	*±0.01*	*±0.03*

## Data Availability

The original contributions presented in this study are included in the article. Further inquiries can be directed to the corresponding author.
